# Effect of Temperature on Mechanical Behavior of Concrete Reinforced with Different Types of GFRP Bar

**DOI:** 10.3390/polym14173437

**Published:** 2022-08-23

**Authors:** Ruan Carlos de Araújo Moura, Paulo Roberto Lopes Lima, Daniel Véras Ribeiro

**Affiliations:** 1 Post-Graduate Program in Civil Engineering (PPEC), Federal University of Bahia, Salvador 40210-630, BA, Brazil; 2 Department of Exact and Technology Sciences, State University of Santa Cruz, Ilhéus 45662-900, BA, Brazil; 3 Department of Technology, State University of Feira de Santana, Feira de Santana 44036-940, BA, Brazil; 4 Department of Materials Science and Technology, Federal University of Bahia, Salvador 40210-630, BA, Brazil

**Keywords:** GFRP bars, durability, thermal degradation

## Abstract

Glass fiber reinforced polymer (GFRP) bars have been increasingly used as reinforcement in concrete structures. However, when the bars are exposed to high temperatures, there is a change in the internal structure of the polymer which affects the tensile strength of the matrix and its adhesion with the fibers, reducing the mechanical strength of the bar. In addition, with increasing temperature, the bar-concrete interface is also damaged by the decomposition of hydration products from the cement paste and the loss of surface adhesion. The intensity of these changes is associated with the type of resin used as a matrix since each polymer has its own molecular structure that provides a greater or lesser ability to resist the changes imposed by temperature. The present study evaluates the mechanical behavior of reinforced concrete containing different types of GFRP bars and subjected to temperatures of 150 °C, 300 °C, and 350 °C. The GFRP bars with three types of matrices (polyester, vinyl ester, or epoxy) were mechanically evaluated under tension in two conditions: isolated and inserted into reinforced concrete specimens with a thickness of 20 mm, using two types of concrete (with and without silica fume). Degradation mechanisms at the bar/concrete interface were evaluated using thermogravimetric analysis (TGA), differential scanning calorimetry (DSC), differential thermal analysis (DTA), and bond techniques. The results showed that the type of matrix has a significant influence on the tensile behavior of GFRP bars, with the epoxy matrix showing the best performance, followed by bars with vinyl ester and polyester matrix resins. The use of silica fume improved the performance of the concrete coating and, consequently, improved the protection of GFRP bars, hindering the diffusion of oxygen and heat; bar/concrete adhesion was compromised by thermal degradation of GFRP bar ribs.

## 1. Introduction

The glass fiber reinforced polymer (GFRP) bar is a structural construction material that is attracting considerable attention due to its physical, chemical, and mechanical characteristics, such as electrochemical corrosion resistance, high strength-to-weight ratio, low thermal conductivity, electromagnetic transparency, and ease of manufacture, transport, and handling [1]. Compared to GFRP bars, carbon fiber reinforced (CFRP) bars have higher mechanical strength and greater chemical resistance to an alkaline environment [2]. However, due to its lower cost, the GFRP bar has become more used in Civil Engineering. The higher electrolytic resistance, in comparison to CFRP bars, has also motivated the use of bars with fiberglass, since the main motivation for the application of FRP bars in reinforced concrete structures is to avoid corrosion of the reinforcement, common in steel bars, and with a great impact on the maintenance cost of buildings and special structures, such as bridges, ports [2,3,4].

However, one of the main concerns regarding the use of FRP bars refers to the effect of temperature on their mechanical properties, since polymer matrices, mainly based on thermoset polymers (vinyl ester or epoxy) [5], undergo changes in their intermolecular structure when exposed to temperatures greater than the glass transition temperature.

The polymer molecules absorb thermal energy, which may weaken the intermolecular interactions and subsequently break the chemical bonds in the main chain of the polymer. Changes in color and texture have been verified in bars as a function of temperature and time of exposure to high temperature, which has been attributed to thermal decomposition of the polymer matrix [6] due to oxidation and the generation of anhydrides [7]. Glass fibers have better thermal properties compared to the matrix and continue to support loads in the longitudinal direction up to temperatures close to 980 °C [8], however, damage to the polymer matrix due to the increase in temperature results in the irreversible loss of the ability to transfer forces and protect the glass fibers.

The effects of heating are propagated to the bottom of the bar, resulting in damage to the fiber/matrix interface and properties of the glass fibers with consequent loss of the mechanical properties of the GFRP bars. Recent studies have evaluated the properties of GFRP bars after exposure to high temperatures indicating a reduction in tensile strength of up to 40% [9,10,11] depending on the type of polymer, temperature and time of exposure to heating.

In reinforced concrete structures, the damage to the GFRP bar due to the action of external agents, including the increase in temperature, is minimized by protecting the concrete cover, as observed by Alsayed et al. [6], Najafabadi et al. [12] and Saaf [13]. The concrete cover is established by standards [14,15,16] and can vary from 20 to 60 mm, depending on the environmental exposure class. The Canadian standard [14] establishes a procedure for the determination of concrete cover for a required fire resistance rating since the fire resistance of FRP reinforced concrete depends on the critical temperature of FRP reinforcement, the thickness of the concrete cover, and the type of concrete used.

When exposed to high temperatures, the cement paste of concrete undergoes changes in its chemical structure, with a reduction in mechanical strength and modulus of elasticity, which depends on the composition of the concrete. The presence of silica fume, for example, allows for greater resistance of the concrete to the action of high temperatures [17]. As a consequence of the degradation of the cement paste due to high temperatures, the GFRP bar-concrete interface is altered, modifying the adhesion and stress transfer with increasing temperature [18,19,20]. Ellis et al. [18] reported a 4% to 73% reduction in the bond stress of GFRP bars (manufactured with vinyl ester resin) and concrete when exposed to temperatures ranging from 100 °C to 400 °C. Solyom et al. [19] investigated the adhesion of vinyl ester matrix GFRP bars to concrete when exposed to temperatures of 80 °C, 165 °C, 190 °C, and 300 °C, and observed a reduction in the adhesion of 32.6%, 46.0%, 68.7%, and 93.0%, respectively. Rosa et al. [20] evaluated the adhesion of GFRP bars to concrete when exposed to temperatures ranging from 20 °C to 300 °C and observed reductions in adhesion of up to 90%.

These results have raised concerns about the structural performance of structural elements reinforced with GFRP bars exposed to high temperatures since most of a bar’s mechanical performance can be lost in the early stages of a fire. Exposure to fire is one of the most aggressive situations to which structures can be subjected during their useful life. Usually, building structures are protected by other protective measures, but as the fire progresses, the structural integrity needs to be maintained until the building is completely evacuated and the fire is extinguished. Thus, the knowledge of the effect of temperature on the behavior of concrete structures reinforced with FRP bars is fundamental for Civil Engineering.

Thus, in order for GFRP bars to be used safely, in addition to the basic mechanical properties, it is necessary to determine the fire resistance of the bars, insulated or embedded in concrete, mainly due to the different types of polymeric matrices that can be used in the manufacturing of GFRP bars due to modifications of concrete with the addition of pozzolanic materials. The aim of this study is to analyze the effect of the type of polymer matrix used in the manufacture of GFRP bars and the effect of the type of concrete on the mechanical behavior and bond strength of reinforced concrete subjected to temperatures of 150 °C, 300 °C, and 350 °C. Degradation mechanisms at the bar/concrete interface were evaluated using thermogravimetric analysis (TGA), differential scanning calorimetry (DSC), and differential thermal analysis (DTA) techniques.

## 2. Materials and Methods

### 2.1. Materials

#### 2.1.1. GFRP Bars

**Figure 1:**
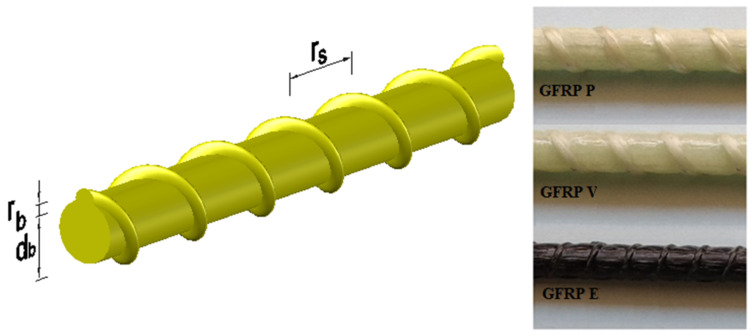
Geometric characteristics of GFRP bars.

For the development of this study, GFRP bars were used with three types of matrices: isophthalic polyester (GFRP-P), vinyl ester (GFRP-V), and epoxy (GFRP-E). The nominal diameter of the bars used was 13 mm, with helical ribs, as shown in Figure 1. The bars were produced by pultrusion, with reinforcement consisting of unidirectional continuous glass fiber filaments (rovings); GFRP-P and GFRP-V bars with the E-type glass fiber and GFRP-E bars with the ECR-type glass fiber. Table 1 shows the physical properties and geometric characteristics of GFPR bars.

#### 2.1.2. Concrete

For the production of reinforced concrete elements, two types of concrete were produced (conventional concrete and concrete containing silica fume) in order to verify the effect of the addition of pozzolan on the protection of the GFRP bars.

A high initial strength cement was used with a specific mass of 3.13 g/cm^3^, a BET surface area of 1.4 m^2^/g, and an average equivalent diameter (D_50_) of 15.0 µm. The silica fume had a specific mass (Micromeritics Accupyc 1340 helium gas pycnometer, Norcross, GA, USA) of 2.32 g/cm^3^, a BET (Micromeritics model Gemini VII, Norcross, GA, USA) surface area of 15.2 m^2^/g, and an equivalent diameter (D_50_), determined by laser granulometry (CILAS 1180 particle-size analyzer, Orleans, France), equal to 43.0 µm. Table 2 shows the properties of the aggregates (sand and gravel) and the respective characterization methods used.

The reference concrete mix (REF) was defined based on the method proposed by the American Concrete Institute (ACI). A mass ratio of 1:2.50:2.40:0.65 was defined (cement + silica fume: sand: gravel: water), with the dimension maximum of the gravel being 9.5 mm, the silica fume content varying between 0% (REF) and 10% (SA-10), and replacing the cement content of 425.8 kg/m^3^. The slump in the truncated cone was 170 ± 20 mm to maintain workability during the molding of the specimens.

The physical-mechanical properties of the concrete produced are shown in Table 3.

Note that the use of silica fume increased the compressive strength and the apparent specific weight of the concrete, reducing the open porosity of the material. No significant differences were observed in the diametral compressive strength and in the elastic modulus of the evaluated concretes.

### 2.2. Conditioning of Specimens

The GFRP bar specimens were tested at 23 °C and at high temperatures (150 °C, 300 °C, and 350 °C), above the glass transition temperatures (T_g_) and close to the decomposition temperature of the polymeric matrix (T_d_). The specimens were heated at a rate of 10 °C/min until the target temperature, remaining at this level for 30 min [33].

### 2.3. Methods

#### 2.3.1. Thermal Analysis

Thermogravimetric analysis (TGA/DTG), differential thermal analysis (DTA), and differential scanning calorimetry (DSC) were performed on the GFRP bars and cement paste, and a mass ratio of 1:0.65 was defined (cement + silica fume: water), with a silica fume content varying between 0% (REF) and 10% (SA-10), replacing the mass of cement.

A TA Instruments SDT Q600 thermogravimetric analyzer (New Castle, DE, USA), using approximately 10 mg per sample was used in the analysis. The tests were performed according to the procedures described in ASTM E1868 [34] and ASTM E1356 [35] standards, for the TGA/DTA and DSC analysis, respectively. The GFRP bar samples were tested in a heating cycle with temperatures ranging between 35 °C and 700 °C, while, in the cement paste samples, the heating cycle temperature varied between 35 °C and 1000 °C, the heating rate was 10 °C/min in a nitrogen atmosphere (purge rate: 10 mL/min).

#### 2.3.2. Tensile Test

**Figure 2:**
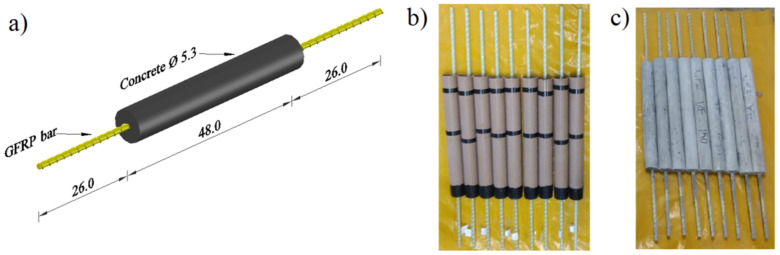
Samples GFRP-Y: (**a**) Dimensions (length in centimeters), (**b**) Molds in PVC tube for cylindric concrete, and (**c**) Samples GFRP-Y formed by GFRP rebar and concrete covered.

Three 1.0 m-long specimens of each type of GFRP bar were prepared and subjected to the tensile test; 27 specimens without the concrete coating (GFRP-N) and 72 specimens with the concrete coating (GFRP-Y), as shown in Figure 2, totaling 99 specimens.

The GFRP bar specimens without concrete coating (GFRP-N) and with the concrete coating (GFRP-Y) were prepared for the tensile test.

1. GFRP bar

An anchoring system had to be used at the ends of these specimens, following the procedures of the ASTM D 7205 standard [23], which recommends that the ends of the GFRP bars be protected, to avoid stress concentration in this region. This anchoring system was formed by an expansive mortar (Split Star, Xiamen, China) and circular steel tube with a diameter of 33 mm and a length of 200 mm.

To maintain the alignment between the GFRP bar and the metal tube, it was necessary to use PVC plastic bushings inside the tube and a wooden frame.

2. GFRP bar with concrete coating

For the preparation of the GFRP-Y specimens, in addition to protecting the ends of the GFRP bars, as described in the previous section, it was necessary to produce and mold the concrete.

The GFRP bars were initially centered in a PVC tube of 48 cm in length and 53 mm in diameter, with the tube being filled with concrete. This procedure allowed the production of a reinforced concrete element with a covering layer 20 mm thick, which is the minimum value required by the design standard. Due to the thickness of the furnace used in the tests, it was not possible to evaluate concrete cover with greater thicknesses.

**Figure 3:**
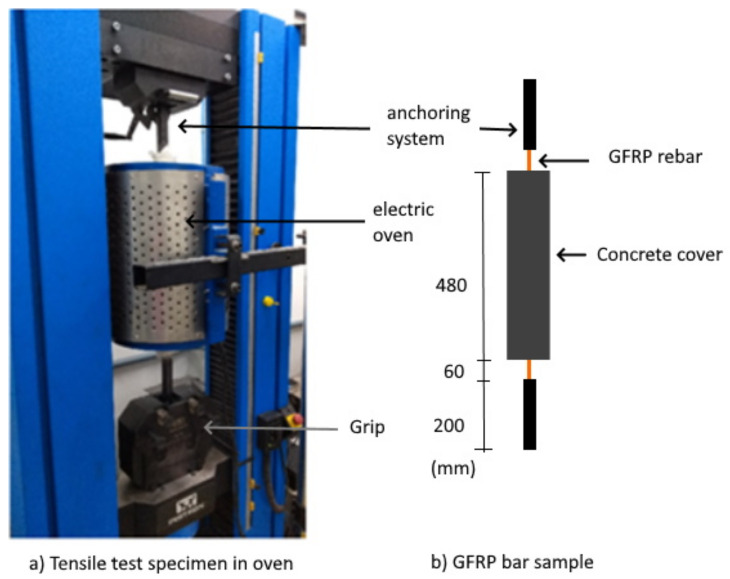
Tensile test setup: (**a**) GFRP bar specimen in oven and (**b**) GFRP bar sample with concrete cover.

After preparing the specimens, the tensile test was performed in a universal machine in an Instron 23-200 (São José dos Pinhais, Brazil), with a load cell of 200 kN, equipped with a vertical electric oven. Initially, the specimens were positioned in this machine (Figure 3a,b). Then, the ends were wrapped in refractory ceramic wool to avoid damaging the equipment and finally, the vertical oven was positioned in the central region of the specimen.

After positioning the specimen, the oven was heated to the desired temperature, remaining at this temperature for 30 min, as described by [33]. Then, the oven was removed to start loading the specimen until it ruptured, with a displacement rate of 2.0 mm/min. The clamps used were kept at room temperature in order to avoid premature rupture mechanisms. The test data were recorded using the software BlueHill Universal 3 (Norwood, MA, USA), linked to the equipment used. The total displacement was measured by the movement of the beam in the testing machine.

#### 2.3.3. Assessment of Bond between GFRP Bar and Concrete

After heating the GFRP-Y specimens to temperatures of 150 °C, 300 °C, and 350 °C, as described in Section 2.3.2, they were subjected to tensile loading up to 50% of the rupture load verified at room temperature. Then, the photographic record was made, to evaluate the distance between cracks, with the aid of the software Image J (1, ImJoy, Bethesda, MD, USA). After unloading, the rods were then loaded until rupture.

The bond strength (*τ_m_*) between the GFRP bars and the concrete was calculated using the value of the mean spacing between cracks and the diametrical compressive strength of the concrete at temperatures of 150 °C, 300 °C, and 350 °C [36]. To determine the tensile strength, cylindrical concrete specimens (10 cm × 20 cm) were heated at a rate of 10 °C/min and kept at the study temperature for 30 min in an oven (Electro Therm Linn, KK -260, Eschenfelden, Germany). Then, these specimens were tested according to standard NBR 7222 [31].

Thus, it was possible to calculate the adhesion tension ($\tau_{m}$), using Equation (1).

(1) $$\tau_{m}=\frac{0.375f_{ct}d_{b}}{\Delta l\;\rho }$$

where $f_{ct}$ is the tensile strength by diametrical compression of concrete (heated at temperatures of 150 °C, 300 °C, and 350 °C), $d_{b}$ is the diameter of the GFRP bar, Δl is the average distance between cracks and ρ is the ratio of the cross-sectional area of the bar and the concrete cylinder.

## 3. Results and Discussion

### 3.1. Thermal Analyses

#### 3.1.1. GFRP Bars

**Figure 4:**
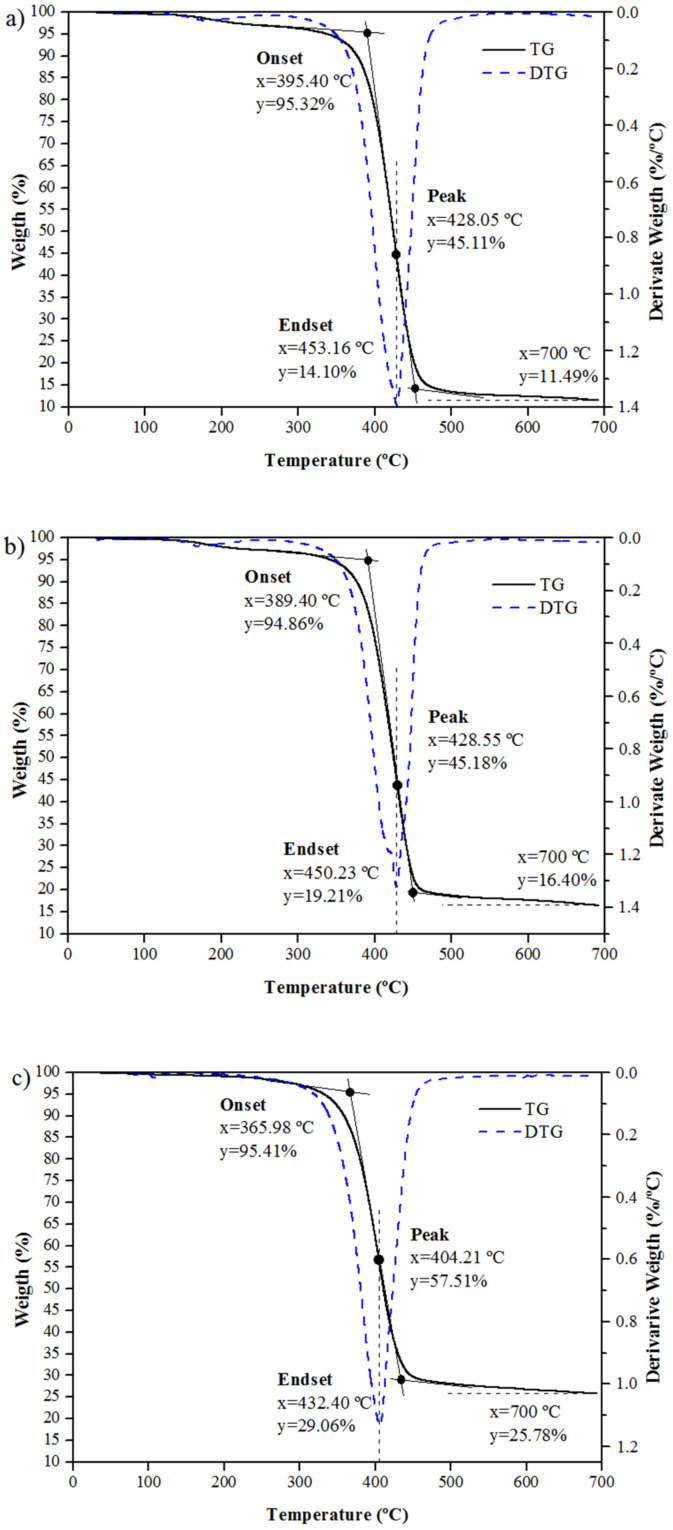
TG and DTG curves of GFRP bars with matrix: (**a**) polyester, (**b**) vinyl ester, and (**c**) epoxy.

The thermogravimetric curves (TG/DTG) of the bar samples with the polyester matrix (P), vinyl ester (V), and epoxy (E) are shown in Figure 4, respectively.

The first mass loss event was observed at about 100 °C, being attributed to the loss of chemically adsorbed water (intermolecular −OH) corresponding to 0.82%, 0.78%, and 0.69% in the GFRP bar samples with the polyester matrix, ester vinyl, and epoxy, respectively. At 300 °C, the mass loss was 3.76% (polyester matrix), 3.46% (vinyl ester matrix), and 2.87% (epoxy matrix), being associated with the breakage of the molecular chains of the polymer matrix [37].

The TG curves showed a sharp slope at temperatures (T_onset_) of 389.4 °C (polyester matrix), 395.4 °C (vinyl ester matrix), and 366.0 °C (epoxy matrix), evidenced by the reduction of the derivative, reaching a well-defined valley in the DTG curve. At the end of this thermal event (T_endset_), mass losses equal to 81.22% (polyester matrix), 75.65% (vinyl ester matrix), and 66.35% (epoxy matrix) were observed and were associated with the thermal degradation of the polymer chains.

At 700 °C, it is possible to observe a residual mass of 11.49% (polyester matrix), 16.40% (vinyl ester matrix), and 25.78% (epoxy matrix), which may be associated with the presence of inorganic components with a higher decomposition temperature. Thus, the GFRP bar sample with the epoxy matrix showed the lowest mass loss, followed by the samples with vinyl ester and polyester matrix, respectively.

**Figure 5:**
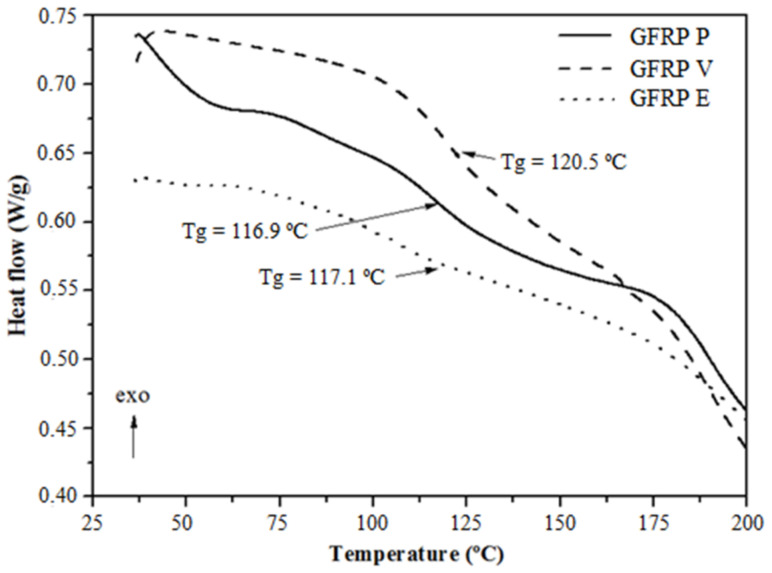
DSC curves of GFRP bar with matrix polyester (P), vinyl ester (V), and epoxy (E).

Figure 5 presents the results obtained by the DSC analysis in samples of the studied GFRP bars. During heating, it is observed that the polymeric matrices show a glass transition at temperatures of 116.9 °C, 120.5 °C, and 117.1 °C for GFRP bar samples with polyester, vinyl ester, and epoxy matrix, respectively. These values are in line with expectations, being higher than the minimum of 110.0 °C established by ASTM D 7957 [38].

The glass transition temperature can be attributed to structural relaxation, since, beyond this temperature, the polymer’s ability to transfer stresses to the glass fibers is greatly impaired, having a negative effect on the tensile strength of GFRP bars. From the TG and DSC analyses, the exposure temperatures of the GFRP bars were chosen at 150 °C (higher value and close to T_g_) and 300 °C (close to the beginning of the decomposition of the polymeric matrix of the GFRP bars).

#### 3.1.2. Cement Paste

**Figure 6:**
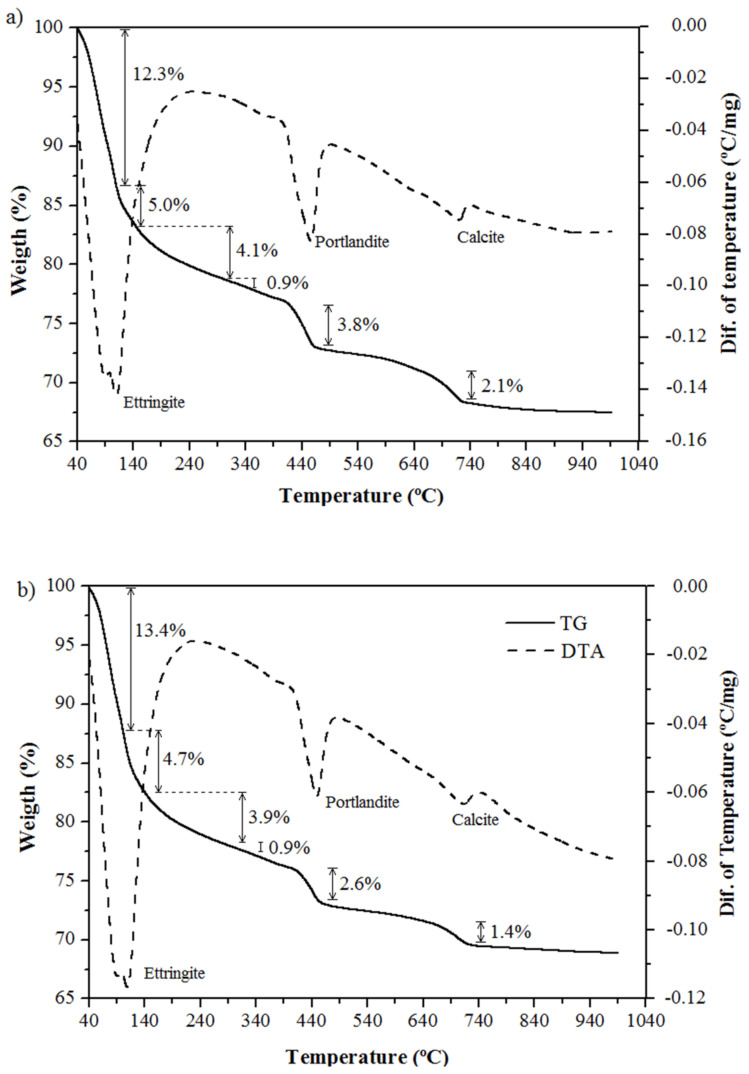
TG and DTA curves of cement pastes: (**a**) REF, and (**b**) SA-10.

To evaluate the effect of exposing the reference cement matrix (REF) and cement containing silica fume (SA-10) to high temperatures, thermogravimetric (TG) and differential thermal (DTA) analyses were performed at 28 days, as shown in Figure 6.

Heating the REF and SA-10 pastes resulted in physical changes with similar thermal behavior. The first mass loss event was identified in the range between 40 °C and 100 °C, representing a loss of 12.3% and 13.4%, respectively, attributed to the effects of ettringite decomposition, which occurs at around 70–90 °C [39].

The second thermal event occurs at 100 °C, with the intensified dehydration of cement pastes due to capillary water (free water), corresponding to 5.0% and 4.7% up to 150 °C, after which the lamellar water evaporates, being adsorbed and chemically associated with hydrated calcium silicate (CSH) crystals, corresponding to 5.0% and 4.8% up to 380 °C [40].

Next, the calcium hydroxide (portlandite) decomposes into lime and water in the temperature range between 380 °C and 460 °C. The water composition of calcium hydroxide corresponds to 3.8% and 2.6%, respectively. These values, when multiplied by 4.11 (ratio of the molecular mass of calcium hydroxide and the molecular mass of water), indicate the remaining content of lime in the sample, which corresponds to 15.6 and 10.7, respectively, indicating that portlandite was consumed in the SA-10 sample. Finally, the calcite decomposes, representing a mass loss of 2.1% and 1.4% for the REF and SA-10 pastes, respectively.

The replacement of 10% of the cement mass by silica fume caused a pozzolanic reaction in the SA-10 cement paste, which reduced the amount of water in the ettringite, hydrated calcium silicate, portlandite, and calcite compounds, thus reducing the mass loss by dehydration. Therefore, the degradation of the cement paste occurred progressively with exposure to heat, which may compromise its mechanical properties. According to these results, it is observed that up to the temperature of 150 °C, there were no significant changes in the cement matrix.

### 3.2. Effect of Temperature on Mechanical Properties

#### 3.2.1. GFRP Bars

**Figure 7:**
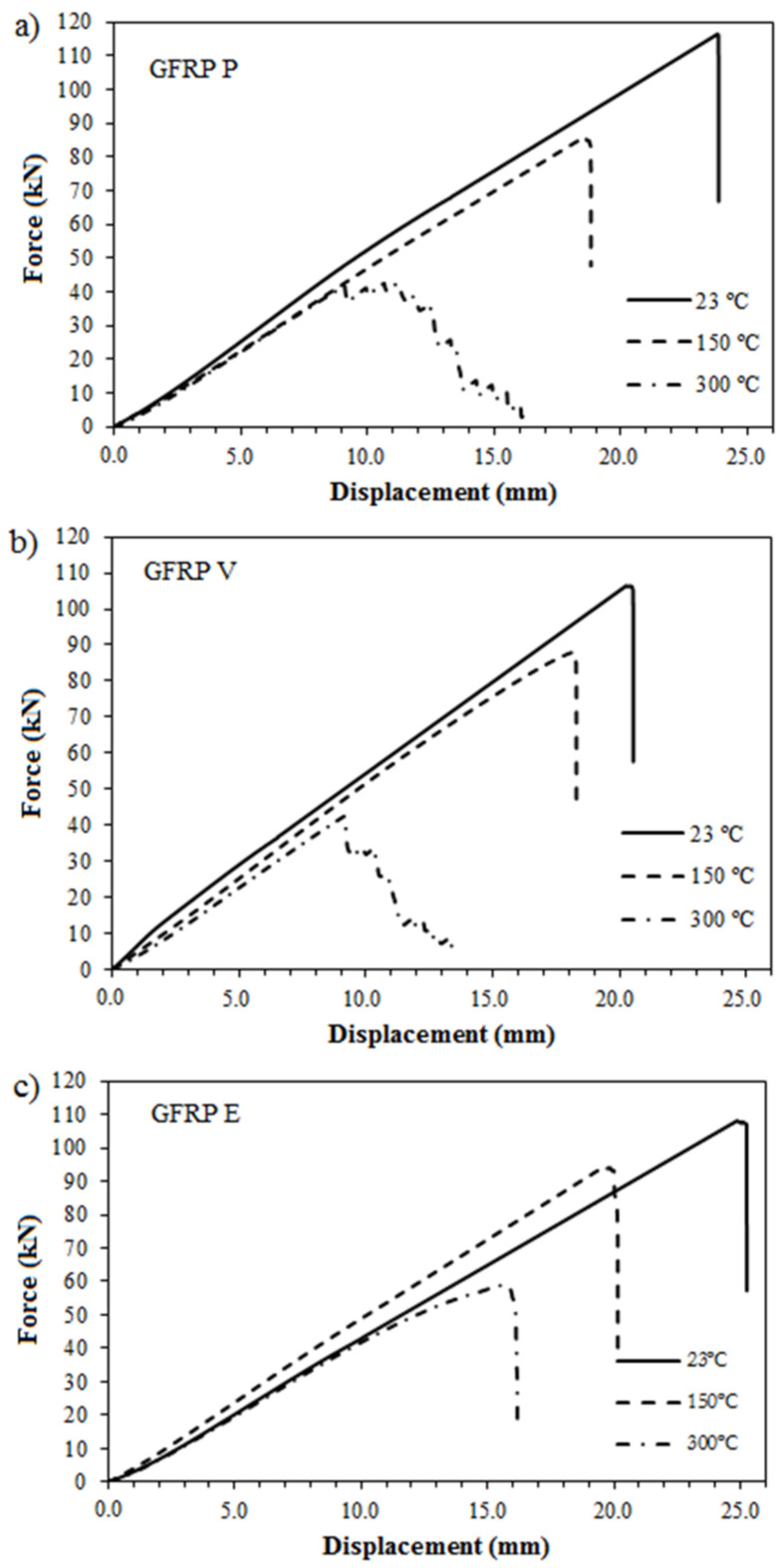
Force vs. displacement curves of samples: (**a**) GFRP P, (**b**) GFRP V, and (**c**) GFRP E.

Figure 7 shows the tensile force-displacement curves of GFRP bars subjected to room temperature (23 °C) and high temperatures (150 °C and 300 °C). It was verified that the bars tested at temperatures of 23 °C and 150 °C exhibited elastic behavior until failure, with brittle failure. For a temperature of 300 °C, a linear stretch to maximum load was observed followed by a gradual loss of strength until failure.

Table 4 presents the tensile strength results of the GFRP bar specimens calculated from the results shown in Figure 7. As observed, the tensile strength of all types of GFRP bars diminished as the exposure temperature increased.

The thermal degradation suffered by the polymer matrix of the GFRP bars causes an increase in the interatomic spacing and the breaking of molecular bonds due to heating. Consequently, the interface between the polymer matrix and the glass fibers was damaged, reflecting a reduction in the transfer of forces between the fibers and the matrix. This loss of mechanical performance can be expressed in terms of the reduction of tensile strength values, as observed in Table 4.

These results corroborate the tensile strength losses reported by Wang et al. [10], who observed that GFRP bars with a polyester matrix (12.7 mm diameter) lost 24%, 42%, 47%, 64%, and 91% of their tensile strength when subjected to temperatures of 100 °C, 200 °C, 300 °C, 400 °C, and 500 °C, respectively, compared to GFRP bars analyzed at room temperature (20 °C). According to Ashrafi et al. [11], the tensile strength of GFRP bars with epoxy matrix subjected to temperatures of 150 °C and 300 °C was reduced by values ranging between 34.5% and 43.3%, respectively, in relation to the specimens tested at room temperature.

**Figure 8:**
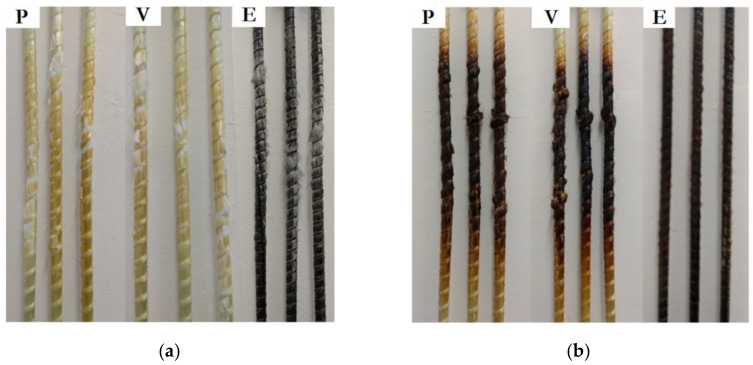
Failure modes of GFRP bars tested at temperatures of (**a**) 150 °C, and (**b**) 300 °C.

Figure 8 shows the appearance of the bars after testing. A color change was observed on the surface of the bars tested at 150 °C (above the T_g_ of the polymer matrix). As the temperature increased to 300 °C, this color change intensified, and heat and toxic volatiles were released due to the thermal degradation of the polymer matrix [9].

#### 3.2.2. Concrete Reinforced with GFRP Bars

**Figure 9:**
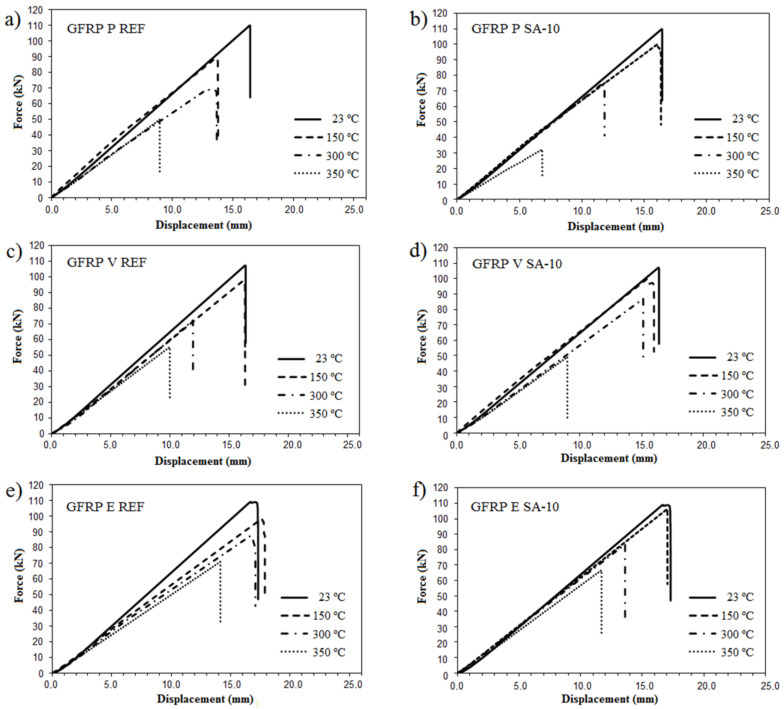
Relation between the force vs. displacement for samples of: (**a**,**b**) GFRP P, (**c**,**d**) GFRP V, and (**e**,**f**) GFRP E, for concrete REF and SA-10, respectively.

Figure 9 shows the force-displacement behavior under direct tension of reinforced concrete with GFRP bars, using reference concrete (Figure 9a,c,e) and with silica concrete (Figure 9b,d,f), subjected to temperatures of 150 °C, 300 °C, and 350 °C. From the maximum load value, and using the cross-sectional area of the bar, it was possible to calculate the tensile strength, as shown in Table 5.

**Figure 10:**
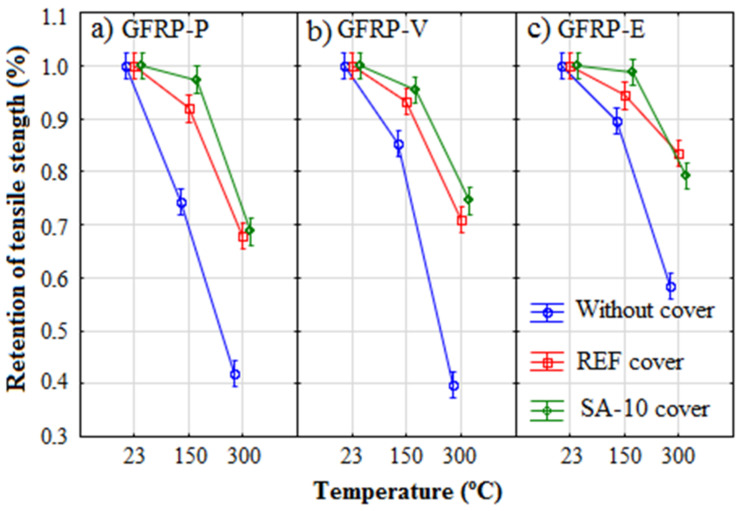
Relation between the retention tensile strength and the test temperature for samples of: (**a**) GFRP P, (**b**) GFRP V, and (**c**) GFRP E.

As observed in insulated bars, there is a reduction in the strength of concrete reinforced with GFRP bars with the increase in temperature. However, due to the protection that the concrete coating provided the GFRP bars, damage to the tensile strength is less, as shown in Figure 10. For example, while a loss of 60% of tensile strength was observed for the GFRP-P bars subjected to 300 °C, the loss was only 30% with the concrete coating. This was due to the low thermal diffusibility of the concrete, which delayed the contact of heat and oxygen with the GFRP bar [12]. Consequently, the concrete-coated bars (GFRP-Y) did not produce toxic gases and smoke observed in the uncoated bars (GFRP-N).

In an attempt to establish a standard concrete thickness in concrete structures with GFRP bars, the CAN/CSA S806 [15] standard established that the minimum coating thickness must be equal to 3.5 times the diameter of the FRP bar in order to ensure adequate protection. However, this standard does not specify the type of concrete that must be used with regard to its composition.

Concerning the concrete types, heating with the addition of silica fume improves the protection of GFRP bars when compared to reference concrete, particularly at 150 °C. The effect of replacing 10% of the cement mass with silica fume caused a change in the pore structure of the concrete. The reference concrete (REF) presented density and apparent porosity of 2556.0 kg/m^3^ and 18.2%, respectively, while for the SA-10 concrete these values were 2617.0 kg/m^3^ and 16.3, that is, the addition of silica fume promoted a greater barrier to the flow of fluids (heat, air, and water vapor) resulting in greater thermal protection for the GFRP bars.

In addition, the results of the thermogravimetric analysis showed a lower mass loss associated with the decomposition of calcium hydroxide (portlandite) in samples of the cementitious matrix with 10% silica fume, compared to the reference cement matrix, corresponding to 3.8% and 2.6%, respectively. This contributed to the reduction in microcracking of the cement matrix in the concrete samples with silica fume.

The tendency to reduce the mechanical properties of GFRP bars after exposure to high temperatures corroborates the results obtained by several other authors [6,12], who verified a reduction in the tensile strength of GFRP bars subjected to temperatures from 100 °C to 400 °C. Alsayed et al. [6] used a 40 mm thick concrete coating and observed reductions in tensile strength in GFRP bars (12 mm diameter) equal to 3.1%, 12.3%, and 16.8% after exposure to temperatures of 100 °C, 200 °C, and 300 °C, respectively. Najafabadi et al. [12] reported that GFRP bars (10 mm diameter) protected by a 20 mm concrete coating layer experienced a reduction in tensile strength of about 13.4%, 23.2%, and 33.2% after exposure to temperatures of 150 °C, 300 °C, and 400 °C, respectively.

It was observed that specimens heated to 150 °C (higher than T_g_) suffered a reasonable loss of tensile strength (16.9% and 12.2%), which intensified at 300 °C (27.5% and 22.5%), in addition to longitudinal cracks in the concrete specimen. At 350 °C, the longitudinal cracks in the overlay concrete were more evident, with a reduction in tensile strength of around 41.7% and 40.0%.

According to Dwaikat and Kodur [41], heating transforms water into steam inside the concrete and, consequently, causes an increase in pore pressure. When this pressure exceeds the tensile strength of the concrete, it can fracture due to tension in a phenomenon that can be explosive, depending on the heating conditions and the characteristics of the concrete.

**Figure 11:**
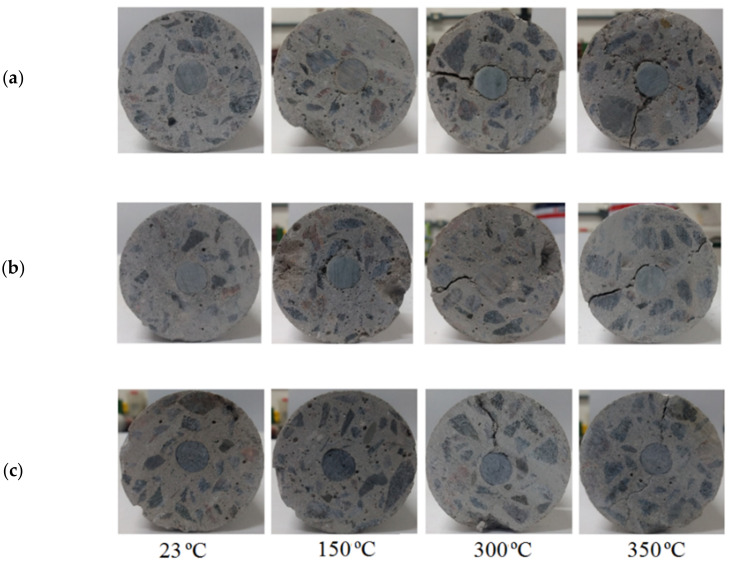
Transversal failure of concrete of samples: (**a**) GFRP P, (**b**) GFRP V, and (**c**) GFRP E at elevated temperatures.

The longitudinal cracks in the GFRP-Y specimens shown in Figure 11 appeared after heating at temperatures of 300 °C and 350 °C due to the difference in thermal expansion in its transverse direction, as the coefficient of thermal expansion of concrete is 7–11 × 10^−6^ °C^−1^ and the coefficient of transverse thermal expansion of a GFRP bar is 21–23 × 10^−6^ °C^−1^ [42,43]. As a result, tensile stresses appeared inside the concrete around the GFRP bars as shown in Figure 11. The appearance of cracks in the concrete coating due to the high transverse coefficient of thermal expansion in the GFRP bars, after exposure to high temperatures, corroborates the results obtained by Aydin [43] and Abdalla [44].

The contribution of different parameters to the ultimate tensile strength of GFRP bars was investigated using a multi-way analysis of variance (ANOVA) (Table 6). The selected output parameter was the final tensile strength of the GFRP bars tested at different temperatures, while the input variables were the type of coating (REF and SA-10), type of GFRP bar (P, V, and E), and the exposure temperature (23 °C, 150 °C, and 300 °C). The effects of coating, bar type, and temperature are statistically significant as the *p*-values are approximately equal to zero.

#### 3.2.3. Adhesion between GFRP Bars and Concrete

According to ACI 440.1R-15 [16], the loss of adhesion between GFRP bars and concrete, due to the increase in temperature, can affect the structural behavior at high temperatures, with an increase in crack openings and deformations in reinforced concrete. Thus, several studies have been carried out to determine the bond stress between GFRP bars and concrete, through the pullout test [18,19,20] or the tie bar test [45,46].

**Figure 12:**
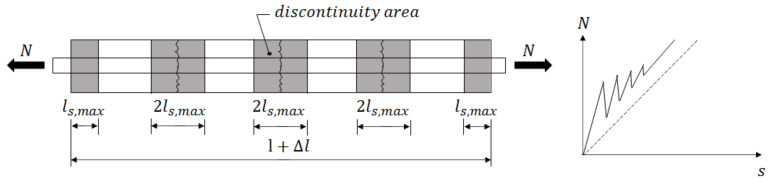
Behavior of a reinforced prismatic bar subjected to imposed deformation. Adapted by [47].

Figure 12 shows the basic behavior of a concrete specimen reinforced with GFRP bars (GFRP-Y) subjected to axial tensile load. Until the appearance of the first crack, the relationship between load and displacement is assumed to be linear and the adhesion efficiency is guaranteed primarily by chemical adhesion between the cement paste and the polymer matrix.

After the first crack is formed, the force transmitted between the two parts of the cracked GFRP-Y specimen was performed only by bond stresses between the GFRP bar and the concrete. With the rupture of the chemical adhesion in the region close to the crack, the mechanical anchoring provided by the ribs is responsible for the largest portion of the adhesion. As the applied loads increase, a new crack forms at a certain distance from the initial crack called the transfer length (l_r_), and strain compatibility is established between the reinforcement and the concrete. New cracks are formed gradually, with relatively uniform spacing (s), in a process called multiple cracking, shown schematically in Figure 12, until the tie beam reaches a stabilized cracking stage and no new cracks are formed. At this stage, the crack spacing is in an interval given by: l_r_ < s < 2l_r_.

**Figure 13:**
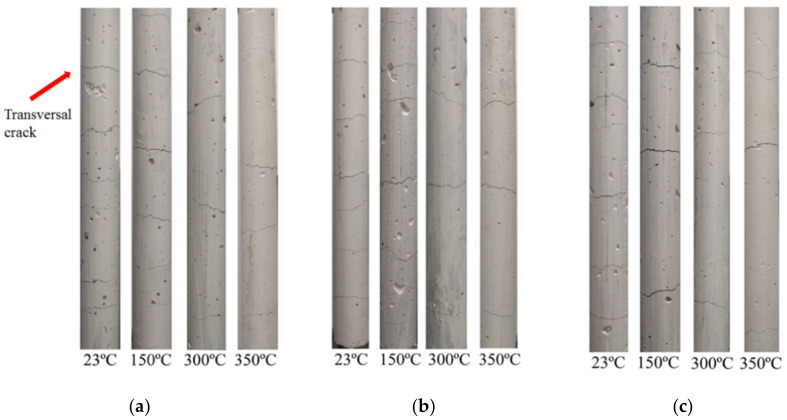
Cracking of a GFRP reinforced ties crack pattern: (**a**) GFRP P, (**b**) GFRP V, and (**c**) GFRP E subjected at 300 °C.

Figure 13 shows the cracking process of the reinforced concrete specimens reinforced with three types of GFRP bars and subjected to different temperatures. As observed, the increase in temperature resulted in a reduction in the spacing between cracks, as shown in Table 6.

Until the appearance of the first crack, the load is supported by the matrix and the reinforcement, together, compromising elastic deformations until the beginning of cracking. With the appearance of the first crack and the process of multiple cracking, there is a gradual degradation of the bond stress between the GFRP bar and the concrete. In the cracking process, the matrix suffers successive losses of strength, to the point where the matrix no longer contributes significantly and the GFRP bar starts to fully support the tensile loads.

With continued application of force, the GFRP bar is pulled to full failure. In addition, changes were observed in the first crack load of the GFRP-Y samples, as shown in Table 7. Moreover, the values of the first crack load in the GFRP-Y-REF specimens are between 3.2 and 2.1 kN, while these values for the GFRP-Y-SA10 specimens are between 6.4 and 2.1 kN.

After the appearance of the first crack, the equilibrium of the GFRP-Y specimen is guaranteed by transferring stresses from one side of the crack to the other through the reinforcing bar. While the bars are subjected only to normal stresses in the cracked region, in the non-cracked region the emergence of shear stresses around the lateral area of the bar allows these stresses to be transferred to the concrete and the equilibrium of the GFRP-Y specimen under axial stresses to be maintained. A section of the uncracked GFRP-Y specimen located at a distance ($l_{r}$) from the first crack was analyzed to establish Equation (2) of equilibrium between the internal forces in the concrete and on the surface of the GFRP bar. Where $A_{C}$ is the cross-sectional area of the concrete.

(2) $$\sigma_{C}A_{C}=\tau A_{lateral}$$

Considering that the lateral area is given by $A_{lateral}=\left(2\pi r\right)l_{r}$ and that the relation between the transversal section of the concrete $\left(A_{c}\right)$ and the transversal section of the GFRP bar $\left(A_{r}\right)$ corresponds to the reinforcement rate, $\rho =\frac{A_{r}}{A_{c}}$, Equation (3) is obtained.

(3) $$l_{r}=\frac{1}{4}\frac{\emptyset }{\rho }\frac{\sigma_{c}}{\tau }$$

where ∅ is the diameter of the GFRP bar and $l_{r}$ is the transfer length.

From Equation (4), it is possible to define the position of a new crack that will occur when the stress in the concrete reaches the tensile strength value, that is, $\sigma_{C}=f_{ct}$ and to determine the likely spacing between cracks after the multiple cracking phase. According to Schlicke et al. [48], the new crack will occur at a distance in the interval between $l_{r}$ and $2l_{r}$, as shown in Equation (4):

(4) $$0.25\frac{\emptyset }{\rho }\frac{f_{ct}}{\tau_{min}}<s<0.5\frac{\emptyset }{\rho }\frac{f_{ct}}{\tau_{max}}$$

Based on this concept, NBR 7477 [36] establishes that the average spacing between cracks in a reinforced concrete rod is given by Equation (5).

(5) $$s_{m}=0.375\frac{\emptyset }{\rho }\frac{f_{ct}}{\tau_{m}}$$

In another approach, the correlation between crack spacing and bond stress must consider the influence of the concrete coating [49], which results in the addition of a term in the equation, as indicated by the FIB code 2010 [47], according to Equation (6).

(6) $$l_{r}=kc+\frac{1}{4}\frac{\emptyset }{\rho }\frac{f_{ct}}{\tau }$$

where k is an empirical parameter to take into account the influence of concrete coating (as a simplification, c = 1.0 can be used) and c is the coating thickness.

**Figure 14:**
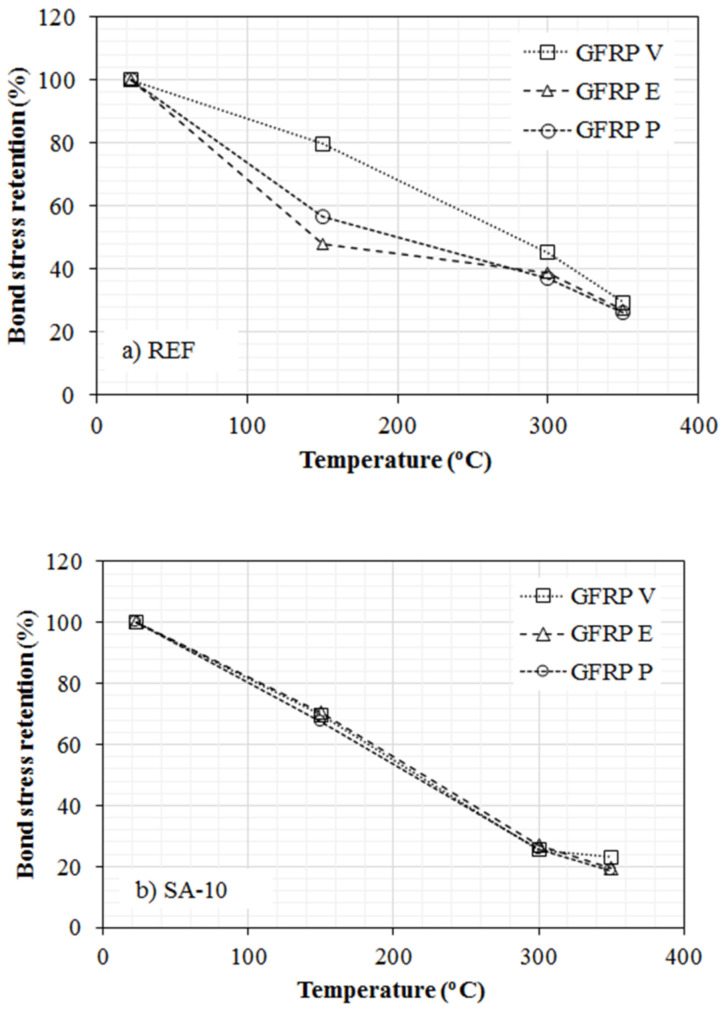
Bond stress retention of GFRP bars with concrete: (**a**) REF, and (**b**) SA-10.

Using the experimental values of crack spacing obtained in this study, it was possible to obtain the average bond strength (Equation (5)) and minimum and maximum values of bond stress, using Equation (4), for the Classical Theory, and Equation (6), for the FIB code 2010 [47]. The results are shown in Table 8 and Figure 14.

It is observed that the reference reinforced concrete specimens (GFRP-Y-REF) heated to 150 °C showed a significant loss in bond strength (43.6%, 20.3%, and 52.0%), which intensified at temperatures of 300 °C (63.1%, 54.9%, and 61.4%) and 350 °C (74.1%, 70.7%, and 72.8%). Similar behavior was observed in the reinforced concrete specimens containing silica fume (GFRP-Y-SA10) heated to 150 °C (loss in bond strength of 32.4%, 30.3%, and 29.3%), 300 °C (loss of 74.3% %, 74.6%, and 73.1%), and 350 °C (81.2%, 76.9%, and 80.4% loss), for GFRP-P, GFRP-V, and GFRP-E bars, respectively.

The heating of GFRP-C specimens caused physical and chemical changes in the concrete, such as dehydration of hydrated products (according to TG/DTA results), and changes to pore structure and microstructure. As a result, there was a reduction in the diametrical compressive strength of the concrete of 16.9% and 12.2% when heated to 150 °C; 27.5% and 22.5% when heated to 300 °C and 41.7% and 40.0% when heated to 350 °C, for concretes REF and SA-10, respectively.

**Figure 15:**
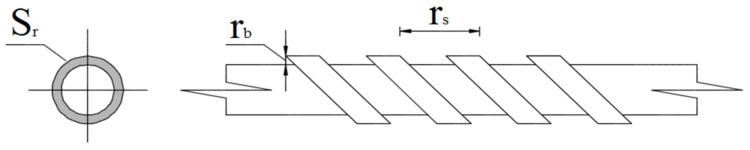
Definition of the area to space ratio ($a_{s}$). Adapted by [49].

In addition to the type of resin, another factor that reduces the adhesion of the GFRP bar with increasing temperature is the difference in its geometry, as shown in Table 1, as the ribs are different. According to Baena et al. [49], it is possible to analyze the influence of the geometry of the GFRP bars on the relative area of the rib ($a_{s}$), which can be calculated through Equation (7) and according to Figure 15.

(7) $$a_{s}=\frac{S_{r}}{r_{s}}$$

where ($S_{r}$) is the projection of the perpendicular area of the rib to the bar axis and ($r_{s}$) is the spacing between the ribs.

Although the specimens of reinforced concrete with GFRP-E bars presented higher tensile strength at high temperatures, this was not observed in their behavior in terms of adhesion strength when compared to specimens reinforced with GFRP-E and GFRP-P bars. The lower bond strength loss of GFRP-V bars is a consequence of their geometric characteristics and the thermal stability of the vinyl ester matrix, with a relative rib area equivalent to 2.81, followed by 2.75 and 2.38 for GFRP-E and GFRP-P, respectively.

The contribution of different parameters to the bond strength of the GFRP-Y specimens was investigated using multifactor analysis of variance, as shown in Table 8. The selected output parameter was the bond strength of the GFRP bars tested in different temperatures, while the input variables were the type of overlay concrete (REF and SA-10), the type of GFRP bar (P, V, and E), and the exposure temperature (23 °C, 150 °C, 300 °C, and 350 °C). It was possible to observe that the effects of temperature and type of concrete versus temperature are statistically significant, with *p*-values approximately equal to zero.

Furthermore, the GFRP-Y specimens with SA-10 concrete presented higher adhesion strength compared to the GFRP-Y specimens with REF concrete due to the reduction of interfacial porosity in the transition zone between the cementitious matrix and the surface of the GFRP bar. The favorable effect on adhesion caused by the addition of silica fume is explained by the fact that these particles filled the areas previously occupied by water in the transition zone. Thus, the densification of the transition zone occurs due to the presence of CSH formed from calcium hydroxide (CH) resulting from the pozzolanic reaction, as shown by the results of the thermogravimetric analysis, which indicated the reduction of the CH content in the cement paste with silica fume.

## 4. Conclusions

In this study, three types of GFRP bar (polyester, vinyl ester, and epoxy matrices) with and without concrete coating were exposed to temperatures of 150 °C, 300 °C, and 350 °C. From the results presented in this work, it is possible to conclude that:

- The tensile strength of GFRP bars decreased significantly when subjected to high temperatures, with 350 °C being the critical temperature at which the decomposition of the polymer matrix occurred, releasing heat and toxic volatiles. GFRP bars exhibited elastic behavior even when heated at temperatures of 150 °C, 300 °C, and 350 °C, although there was a gradual reduction in displacement and consequently, a reduction in the load needed to achieve rupture;
- The 20 mm-thick concrete cladding increased protection against thermo-oxidative degradation resulting from exposure to high temperatures. At the temperature of 350 °C, this protection was compromised by the appearance of longitudinal cracks in the concrete due to the volumetric expansion of the GFRP bars;
- The influence of the GFRP matrix type was significant on the tensile behavior of the bars. In this sense, GFRP bars with epoxy matrix showed the best performance, followed by those with vinyl ester and polyester matrices. Thermoxidative degradation of the GFRP bar ribs resulted in a reduction in the capacity to transfer tension to the concrete, regardless of the type of coating concrete;
- Silica fume improved the performance of the concrete coating and, consequently, improved the protection of GFRP bars, making it difficult for oxygen and heat to diffuse towards the bars. As a result, concrete-coated GFRP bars exhibited better tensile behavior at elevated temperatures compared to GFRP bars that were directly exposed to heat.

## Figures and Tables

**Table 1:** Physical properties and geometric characteristics of GFPR bars manufactured with three types of polymeric matrix: isophthalic polyester (GFRP P), vinyl ester (GFRP V), and epoxy (GFRP E).

Property	Method	GFRP P	GFRP V	GFRP E
Relative density (g/cm^3^)	ASTM D 792:2020 [21]	2.1 ± 0.1	2.0 ± 0.1	1.9 ± 0.1
Fiber content by weight (%)	ASTM D 3171:2015 [22]	82.2 ± 0.1	80.8 ± 0.1	81.2 ± 0.1
Cross-sectional area (mm^2^)	ASTM D 7205:2016 [23]	134.2 ± 0.2	128.2 ± 0.3	112.7 ± 0.1
Tensile strength (MPa)		818.2 ± 10.1	844.8 ± 1.6	971.3 ± 8.6
Bar diameter—*d_b_* (mm)		13.1 ± 0.2	12.8 ± 0.3	12.0 ± 0.1
Rib width (mm)	-	5.1 ± 0.1	5.3 ± 0.2	3.0 ± 0.1
Rib height—r_b_ (mm)	-	1.0 ± 0.1	1.2 ± 0.2	0.8 ± 0.1
Rib spacing—r_s_ (mm)	-	18.6 ± 0.7	18.8 ± 0.7	11.7 ± 0.4

**Table 2:** Material properties of concrete.

Property	Methods	Sand	Gravel
Average particle diameter-D_50_ (mm)	NBR 7211:2009 [24]	0.350	7.100
Specific gravity (kg/m^3^)	NBR NM 52:2009 [25] ^a^, and NBR NM 53:2009 [26] ^b^	2650.0	2770.0
Uncompacted bulk density (kg/m^3^)	NBR NM 45:2006 [27]	1490.0	1390.0
Compacted bulk density (kg/m^3^)		-	1490.0
Fineness modulus	NBR NM 248:2003 [28]	1.6	6.0

^a^ Method used to characterize the sand. ^b^ Method used to characterize the gravel.

**Table 3:** Physical and mechanical properties of concrete at 28 days.

Properties	Methods	REF	SA-10
Compressive strength (MPa)	NBR 5739:2018 [29]	32.7 ± 0.7	38.0 ± 1.3
Elastic modulus (GPa)	NBR 8522:2018 [30]	36.3 ± 1.0	35.1 ± 1.3
Splitting tensile strength (MPa)	NBR 7222:2011 [31]	3.6 ± 0.1	3.6 ± 0.4
Apparent specific gravity (kg/m^3^)	NBR 9778:2009 [32]	2556.0 ± 24.0	2617.0 ± 14.0
Apparent porosity (%)		18.4 ± 1.4	16.3 ± 1.6

**Table 4:** Tensile test results of GFRP bars submitted to elevated temperatures.

Sample	T_g_ ^a^ (°C)	T_d_ ^b^ (°C)	Temperature (°C)	Average Tensile Strength (MPa)	Retention of Tensile Strength (%)
GFRP-P	116.9	389.4	23.0	821.8 ± 7.2	100.0
			150.0	612.6 ± 2.4	74.5
			300.0	338.1 ± 12.9	41.1
GFRP-V	120.5	395.4	23.0	844.8 ± 1.2	100.0
			150.0	720.7 ± 18.4	85.3
			300.0	336.4 ± 11.1	39.8
GFRP-E	117.1	366.0	23.0	971.3 ± 6.1	100.0
			150.0	871.1 ± 17.6	89.7
			300.0	566.7 ± 6.2	58.3

^a^ Glass transition temperature of polymeric matrix. ^b^ Decomposition temperature of polymeric matrix.

**Table 5:** Tensile test results GFRP bars without and with covered concrete (REF and SA-10) submitted at elevated temperatures.

Sample	Temperature (°C)	Average Tensile Strength (MPa)		
		Bar GFRP-N (without Concrete)	Bar GFRP-Y (with Concrete)	
			REF	SA-10
GFRP-P	23.0	821.8 ± 7.2	825.8 ± 12.3	800.0 ± 16.8
	150.0	612.6 ± 2.4	751.8 ± 9.3	796.5 ± 21.4
	300.0	338.1 ± 12.9	555.1 ± 23.1	561.9 ± 16.1
	350.0	-	379.3 ± 32.4	374.6 ± 19.0
GFRP-V	23.0	844.8 ± 1.2	835.6 ± 19.9	840.9 ± 37.5
	150.0	720.7 ± 18.4	787.0 ± 8.4	805.3 ± 5.8
	300.0	336.4 ± 11.1	598.5 ± 20.4	629.1 ± 34.8
	350.0	-	427.9 ± 16.8	405.7 ± 29.1
GFRP-E	23.0	971.3 ± 6.1	953.1 ± 4.4	960.4 ± 5.7
	150.0	871.1 ± 17.6	916.3 ± 27.9	958.0 ± 8.6
	300.0	566.7 ± 6.2	810.0 ± 18.4	769.3 ± 41.5
	350.0	-	582.6 ± 25.3	609.7 ± 23.1

**Table 6:** ANOVA analysis results for GFRP-N and GFRP-Y samples tested at 23 °C–300 °C.

Source of Variation	*SS*	*d_f_*	*MS*	*F*	*p*-Value	Significance
Cover concrete	214,312	2	107,156	184.43	0.00	Yes
GFRP bar	527,409	2	263,705	453.86	0.00	Yes
Temperature	1,284,313	2	642,157	11,105.22	0.00	Yes
Cover concrete−GFRP bar	4850	4	1213	2.09	9.52 × 10^−2^	No
Cover concrete—Temperature	194,243	4	48,561	83.58	0.00	Yes
GFRP bar—Temperature	24,435	4	6109	10.51	2.26 × 10^−6^	Yes
Cover concrete—GFRP bar—Temperature	19,689	8	2461	4.24	5.39 × 10^−4^	Yes
Error	31,375	54	-	-	-	-
Total	2,300,627	80	-	-	-	-

*SS* = the sum of squares of the deviations of all the observations from their mean. *d_f_* = the number of degrees of freedom associated with the sample variance. *MS* = the mean square, which is obtained by dividing the sum of squares by the respective degrees of freedom. *F* = the variation between samples means (Mean Square Between) to the variation within the samples (Mean Squared Error). *p*-value = the probability that an equal amount of variation in the dependent variable would be observed in the case that the independent variable does not affect the dependent variable.

**Table 7:** Bond stress, distance between cracks, and first crack load results GFRP bars with covered concrete at elevated temperatures.

Sample	Temp. (°C)	Distance between Crack (mm)		First Crack Load (kN)		Bond Stress (MPa)	
		REF	SA-10	REF	SA-10	REF	SA-10
GFRP-P	23.0	65.7 ± 4.9	64.4 ± 13.9	2.5 ± 0.1	2.7 ± 0.4	4.2 ± 0.3	4.6 ± 0.9
	150.0	90.7 ± 9.2	85.3 ± 9.2	2.1 ± 0.1	2.4 ± 0.5	2.4 ±0.6	3.1 ± 0.3
	300.0	120.0 ± 0.3	146.7 ± 23.1	2.5 ± 0.3	2.6 ± 0.2	1.6 ± 0.1	1.2 ± 0.2
	350.0	160.0 ± 0.2	186.7 ± 16.2	2.1 ± 0.1	4.1 ± 0.1	1.1 ± 0.1	0.9 ± 0.2
GFRP-V	23.0	74.3 ± 5.7	64.3 ± 10.0	3.1 ± 0.2	2.1 ± 0.1	3.8 ± 0.3	4.3 ± 0.6
	150.0	72.4 ± 6.6	90.7 ± 9.2	2.9 ± 0.8	2.0 ± 0.1	3.1 ± 0.3	3.0 ± 0.3
	300.0	112.0 ± 13.8	160.0 ± 0.8	2.6 ± 0.1	2.7 ± 0.1	1.7 ± 0.2	1.1 ± 0.1
	350.0	160.0 ± 0.5	160.0 ± 0.4	2.0 ± 0.1	6.4 ± 0.7	1.1 ± 0.1	1.0 ± 0.2
GFRP-E	23.0	69.5 ± 10.0	65.7 ± 4.9	3.2 ± 0.1	2.2 ± 0.1	4.4 ± 0.6	4.8 ± 0.4
	150.0	112.0 ± 13.9	85.3 ± 9.2	3.5 ± 0.1	2.2 ± 0.1	2.1 ± 0.3	3.4 ± 0.3
	300.0	120.0 ± 0.5	146.7 ± 23.1	2.3 ± 0.3	3.0 ± 0.3	1.7 ± 0.1	1.3 ± 0.2
	350.0	160.0 ± 0.2	186.7 ± 46.2	2.1 ± 0.1	4.2 ± 0.1	1.2 ± 0.2	0.9 ± 0.2

**Table 8:** ANOVA analysis results for GFRP-Y samples tested at 23 °C–350 °C.

Source of Variation	*SS*	*d_f_*	*MS*	F	*p*-Value	Significance
Type of concrete	0.1301	1	0.1301	1.150	2.89 × 10^−1^	No
GFRP bar	0.1530	2	0.0765	0.676	5.13 × 10^−1^	No
Temperature	123.2830	3	41.0943	363.209	0.00	Yes
Type of concrete−GFRP bar	0.3544	2	0.1772	1.566	2.19 × 10^−1^	No
Type of concrete—Temperature	3.6422	3	1.2141	10.730	1.64 × 10^−15^	Yes
GFRP bar—Temperature	1.1298	6	0.1883	1.664	1.50 × 10^−1^	No
Type of concrete—GFRP bar—Temperature	1.0811	6	0.1802	1.593	1.70 × 10^−1^	No
Error	5.4308	48	0.1131	-	-	-
Total	135.2045	71	-	-	-	-
